# Supplementary material for: High Rate of Transplantation Prior to Review of Status Exception Requests among Adult Heart Transplant Candidates
Source: medRxiv. 2025 Sep 15:2025.09.12.25335606. Preprint. [Version 1] doi: 10.1101/2025.09.12.25335606 (PMC12458605; doi:10.1101/2025.09.12.25335606)
Supplement: Supplement 2 [file media-2.docx]

**
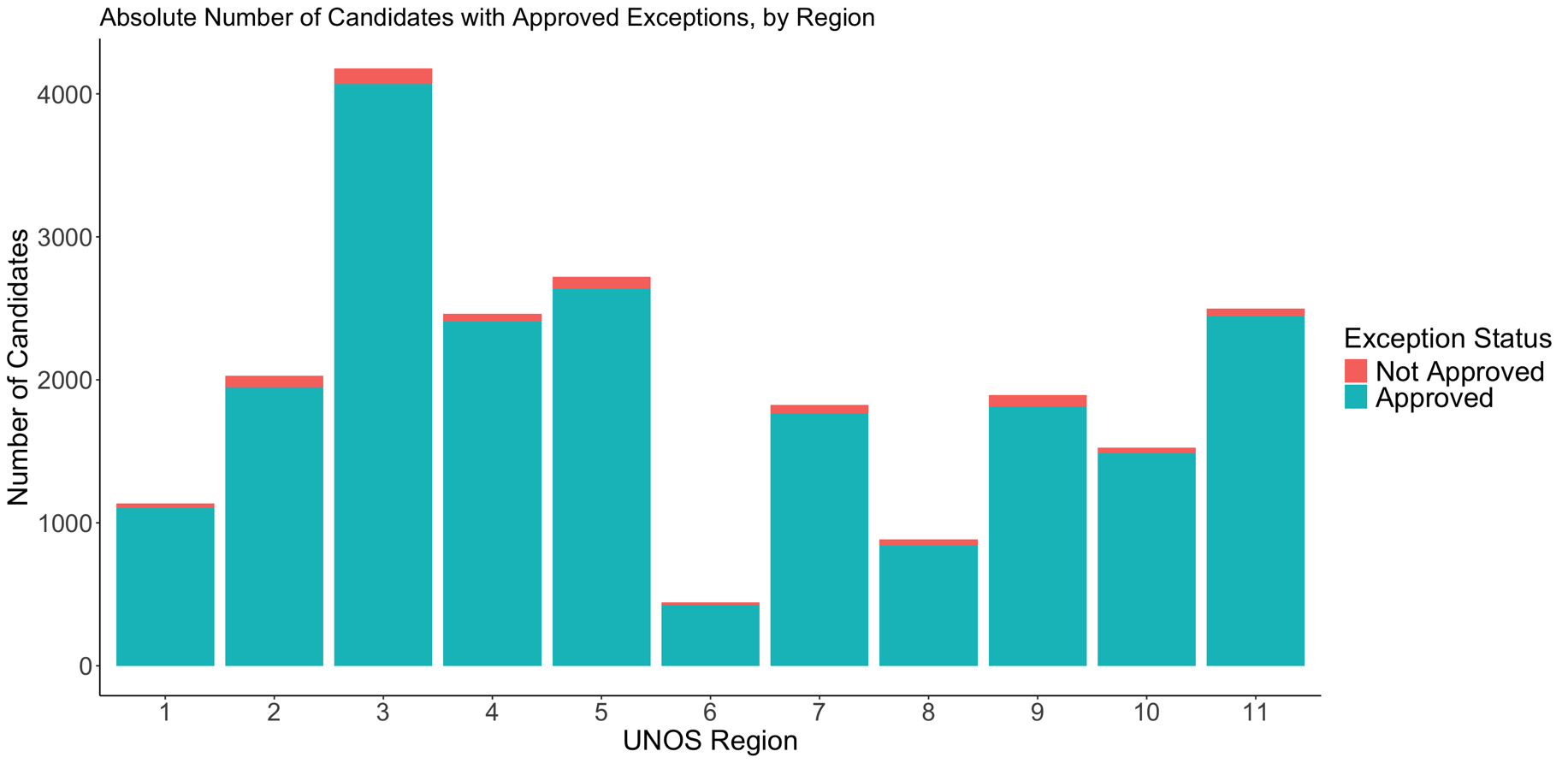
Supplemental Figure 2**: Absolute number of adult heart transplant candidates with approved exception applications, stratified by UNOS region
