# Supplementary material for: High Rate of Transplantation Prior to Review of Status Exception Requests among Adult Heart Transplant Candidates
Source: medRxiv. 2025 Sep 15:2025.09.12.25335606. Preprint. [Version 1] doi: 10.1101/2025.09.12.25335606 (PMC12458605; doi:10.1101/2025.09.12.25335606)
Supplement: Supplement 4 [file media-4.docx]

**
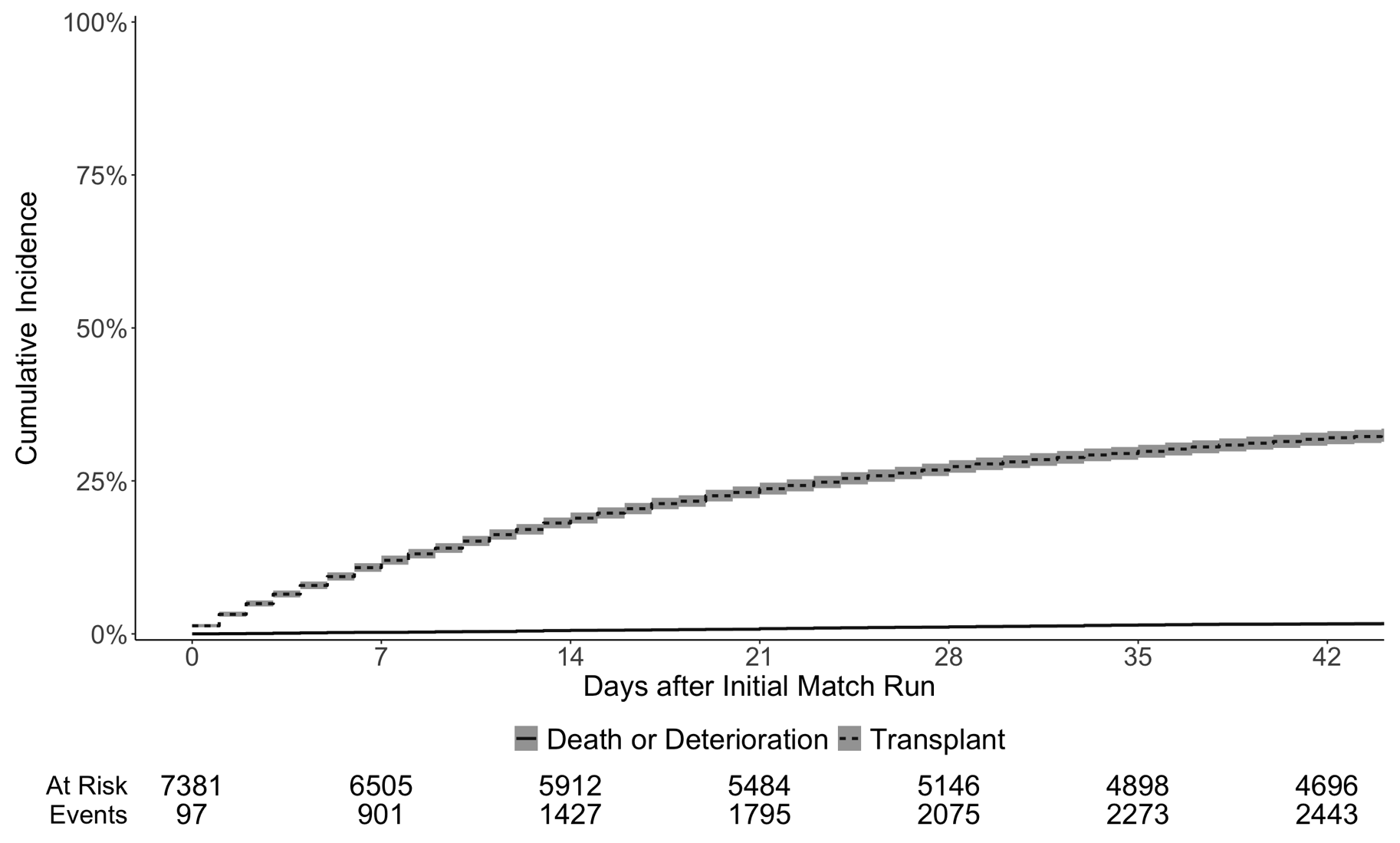
Supplemental Figure 4**: Cumulative incidence within 6 weeks of death or removal for clinical deterioration, treating transplantation as a competing event, of potential transplant recipients bypassed by the 115 candidates who obtained heart transplants with status exceptions that were eventually denied by the regional review boards.
